# Author Correction: Designing minimal genomes using whole-cell models

**DOI:** 10.1038/s41467-020-15994-3

**Published:** 2020-05-06

**Authors:** Joshua Rees-Garbutt, Oliver Chalkley, Sophie Landon, Oliver Purcell, Lucia Marucci, Claire Grierson

**Affiliations:** 1 BrisSynBio, University of Bristol, Bristol, BS8 1TQ UK; 2 School of Biological Sciences, University of Bristol, Bristol Life Sciences Building, 24 Tyndall Avenue, Bristol, BS8 1TQ UK; 3 Department of Engineering Mathematics, University of Bristol, Bristol, BS8 1UB UK; 4 Bristol Centre for Complexity Science, Department of Engineering Mathematics, University of Bristol, Bristol, BS8 1UB UK; 5 Engine Biosciences, MBC Biolabs, 733 Industrial Road, San Carlos, CA 94070 USA; 6 School of Cellular and Molecular Medicine, University of Bristol, Bristol, BS8 1UB UK

**Keywords:** Computer modelling, Genomic engineering, Synthetic biology

Correction to: *Nature Communications* 10.1038/s41467-020-14545-0, published online 11 February 2020.

The original version of this Article contained three errors in Fig. 4, in which the GAMA genome was erroneously labelled GAMA_236, the gene MG_270 was erroneously listed as different between Minesweeper and GAMA, and the gene MG_270 erroneously appeared in dark blue in the Minesweeper genome and was erroneously absent in the GAMA genome. This has been corrected in both the PDF and HTML versions of the Article.

