# Supplementary material for: Designing minimal genomes using whole-cell models
Source: Nat Commun. 2020 Feb 11;11:836. doi: 10.1038/s41467-020-14545-0 (PMC7012841; doi:10.1038/s41467-020-14545-0)
Supplement: Supplementary file 2 — Supplementary Information [file 41467_2020_14545_MOESM2_ESM.pdf]

Designing Minimal Genomes Using Whole-Cell Models

Rees-Garbutt and Chalkley et al.

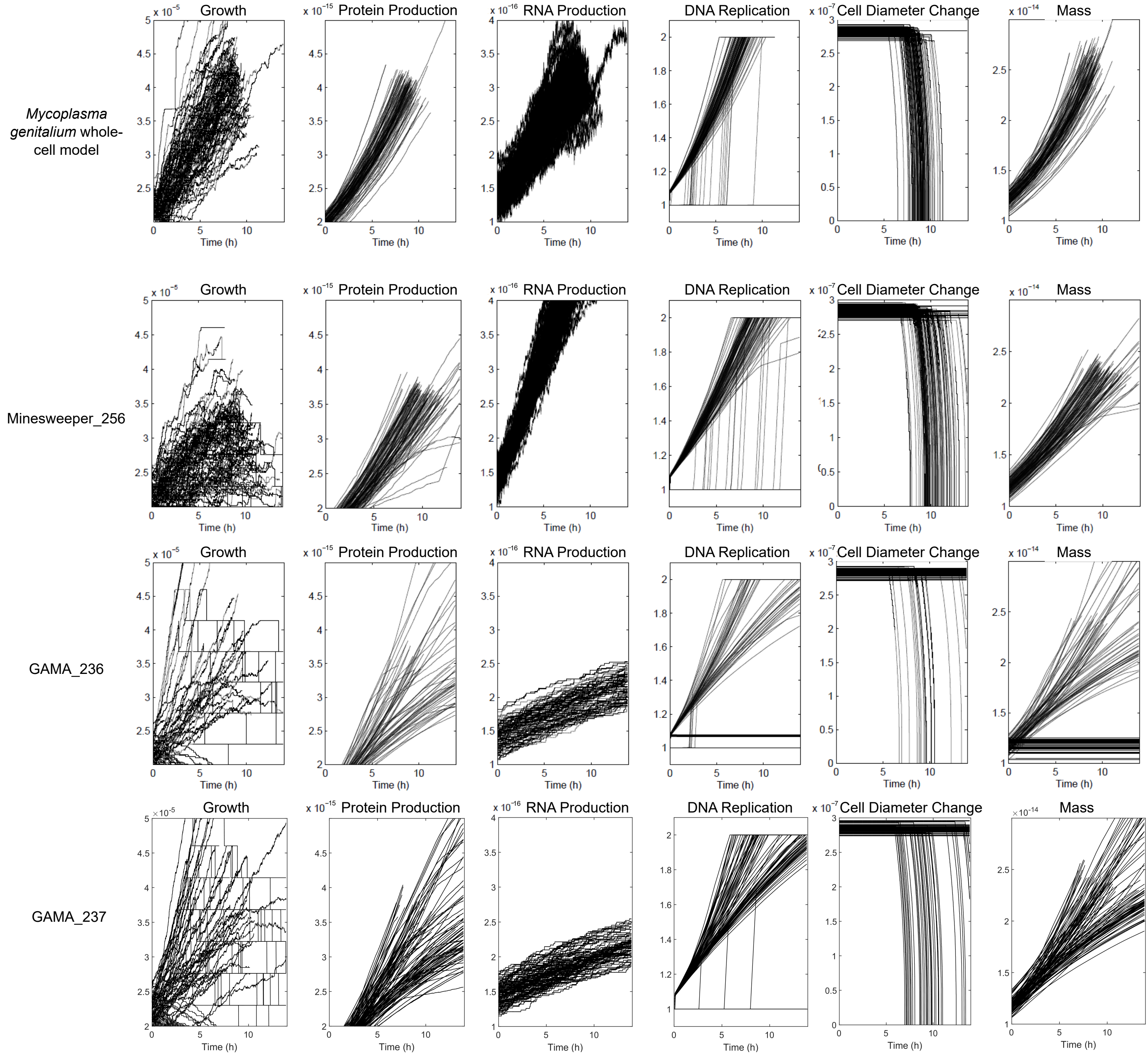

Supplementary Figure 1. Comparison of unmodified *Mycoplasma genitalium* whole-cell model, Minesweeper_256, GAMA_236 and GAMA_237 outputs

100 in-silico replicates, with second-by-second values plotted for 6 cellular variables over 13.89 hours (the default endtime of the simulations).

The top row shows the expected cellular behaviour (previously show by Karr et al 18) and is used for comparison. Minesweeper_256, GAMA_236, GAMA_237 show deviations in phenotype caused by gene deletions. Non aggregated data for each in-silico simulation is available (see Methods).
