# Supplementary material for: Designing minimal genomes using whole-cell models
Source: Nat Commun. 2020 Feb 11;11:836. doi: 10.1038/s41467-020-14545-0 (PMC7012841; doi:10.1038/s41467-020-14545-0)
Supplement: Supplementary file 3 — Description of Additional Supplementary Files [file 41467_2020_14545_MOESM3_ESM.pdf]

Description of Additional Supplementary Files

File Name: Supplementary Data 1
Description: Single gene knockout analysis

File Name: Supplementary Data 2
Description: Inconsistent Genes / Binomial Proportion Confidence interval

File Name: Supplementary Data 3
Description: This Paper vs Karr et al. 2012

File Name: Supplementary Data 4
Description: Decision tree

File Name: Supplementary Data 5
Description: Minesweeper_256 + GAMA_236 / 237 Comparison

File Name: Supplementary Data 6
Description: Shared and Unique Gene Deletions of Minesweeper_256 and GAMA_237

File Name: Supplementary Data 7
Description: Mycoplasma genitalium whole-cell model + MG_006 + Minesweeper_256 + GAMA_236 + GAMA_237

File Name: Supplementary Data 8
Description: Investigation of a potential redundant essential pair and group

File Name: Supplementary Data 9
Description: Matching Genes and Go (Biological Process) Terms for Mycoplasma genitalium whole-cell model

File Name: Supplementary Data 10
Description: Go Terms Dictionary

File Name: Supplementary Data 11
Description: Matched Genes and GO Terms

File Name: Supplementary Data 12
Description: Minesweeper_256 Deletions Impact on GO Terms

File Name: Supplementary Data 13
Description: GAMA_237 Deletions Impact on GO Terms

File Name: Supplementary Data 14
Description: Theory underlying GAMA genome design algorithm

File Name: Supplementary Data 15
Description: Investigating a stubborn gene

File Name: Supplementary Data 16
Description: Improving division rate

File Name: Supplementary Data 17
Description: Reactions that produce ATP in the M.g whole cell model

File Name: Supplementary Data 18
Description: Shared genes vs JCVI-Syn3.0.
